# Mortality in Paris, London, Lyons, and Chicago

**Published:** 1874-11-01

**Authors:** 


					﻿UOitorial ‘Departnient,
TT is said that when a disease is
once recognized, it is half cured.
This is rather a consoling maxim,
and it is almost the sole consolation
left us when we survey the record of
deaths among the children of this
city during the hot season, just
past,
We have compared the mortality
statistics of Paris, London, Lyons,
and Chicago in the appended table,
for the month of July, since that is
the month during which the fatality
from cholera infantum is, with us, the
greatest. We are unable to specify
the precise percentage of deaths from
this disorder in each city, since chol-
era infantum is not tabulated in all of
the foreign bulletins, but the aggre-
gate of fatality from bowel affections
in summer, must be largely due to in-
fantile disorders.
However humiliating it may be to
confess our weakness in this particu-
lar, it is certainly well to recognize it.
The story told by the accompanying
table bears its own moral, and sug-
gests a problem, whose solution is in-
cumbent upon every medical man in
Chicago:
Mortality in the Cities of Paris, London, Lyons, and Chicago, for July, 1874.
Mortality of Special	Disorders.	£	o
----------------------------------------o	■- "
6 g .	2	•	« 2
«.2 o-g
CIT1ES- .	i i id I ?l
|	« i s	J If? £ t2 til
rt	”2	"	B’S3	. c S' c c 3
O	rf	O	E JU U	u	O O — O1
CL	J	J	cnS tziHMMOa OHCLCU
PARIS_______ 1,851,792	48,50,112,20,	09	2	39	6	69	43	76	25	72	25602902 -0015 .000038
N.	E.
LONDON......	3,400,701	51,30,490,65,	48	4	99	141	| 72	30	325	45	356	4015 5087-0014 .000104
. N.	W.
LYONS_______	323,954	45,45,464,49,	25	o	5	2	11	6	7	9	85	483 608.0018 .00026
N.	E.
CHICAGO	.—	53I,7I3	41,54,00 87,	38	7	4	12	10	4	6	4	767	640 1454 .0027	.0014
N.	W.
				

